# Phase Equilibria Related to NiGa_5_ in the Binary Ni-Ga System

**DOI:** 10.3390/ma17040883

**Published:** 2024-02-14

**Authors:** Chih-Chia Bill Chang, C. R. Kao

**Affiliations:** 1 Department of Materials Science and Engineering, National Taiwan University, Taipei 106216, Taiwan; d08527007@ntu.edu.tw; 2 Advanced Research Center for Green Materials Science & Technology, National Taiwan University, Taipei 106216, Taiwan

**Keywords:** phase diagram, phase equilibria, intermetallics, EPMA, XRD, NiGa_5_

## Abstract

The assembly of Ga alloys with Ni or Ni alloy has been widely developed for various low-temperature applications in recent years. In the constituent Ni-Ga binary system, however, the phase equilibrium with the phase “NiGa_5_” and its stability has scarcely been investigated. The present study used the diffusion couple technique combined with SEM-EPMA and XRD analysis to examine the phase stability and the homogeneity range of the phase. The results show that “NiGa_5_” is a stable phase in the binary system with little homogeneity range and suggest that the peritectic reaction $L+{\mathrm{Ni}}_{3}{\mathrm{Ga}}_{7}\to {\mathrm{NiGa}}_{5}$ lies between 112.0 and 115.5 °C. This work provides new information for the modification of the Ga-rich low-T region of the Ni-Ga phase diagram.

## 1. Introduction

In recent years, the Ni-Ga system has gained significant attention due to growing interest in utilizing gallium and its alloys in conjunction with nickel or nickel alloys. Gallium alloys, characterized by liquidus temperatures lower than room temperature, have found applications in soft robotics and wearable devices owing to their inherent electrical conductivity and fluidicity. For example, Suin Kim et al. [1] and Kadri Bugra Ozutemiz et al. [2] incorporated the Electroless Nickel Immersion Gold (ENIG) finishing of electronic chips in contact with gallium alloys to fabricate flexible electronics with liquid metal circuits. Biao Ma et al. [3] dispersed Ni micro-particles in gallium alloys and magnetically patterned the conductive mixture. For the novel idea of non-thermal joining, pure gallium was utilized in transient liquid phase bonding at approximately 30 °C [4,5]. The addition of nickel to the copper substrate accelerated the growth of Intermetallic Compounds (IMCs), effectively reducing the time required to deplete the liquid phase and form a structurally sound joint [4,5,6]. In soldering applications, minor gallium additions in Sn-based solder was intended to enhance wettability, mechanical strength, maximum elongation, and suppress IMC overgrowth [7,8]. Nickel, on the other hand, is often plated on copper substrates to serve as the diffusion barrier layer [9]. Academic resources have extensively studied the rate of Ni or Ni alloy consumption through IMC formation or dissolution when immersed in gallium alloys [10,11,12,13].

In various technological applications, gallium reacts with nickel, forming IMCs during fabrication and service at temperatures ranging from room temperature to the typical tin-soldering temperature of 250 °C. Recently, a gallium-rich Ni-Ga IMC labeled “NiGa_5_” has been proposed in the low-temperature part of the binary system; however, uncertainties persist regarding its composition range, stable temperature, and whether it is a stable phase [14]. Consequently, the present experimental research was carried out to investigate the phase equilibria of the “NiGa_5_” IMC in the Ni-Ga binary system.

## 2. Literature Review

Yamazaki et al. [15] reported the formation of a Ga-rich unknown compound during mechanical alloying of Ni powder and liquid Ga. During this process, “NiGa_4_” is said to be produced by the reaction of this unknown phase and Ni.

Clemens Schmetterer et al. [14] investigated the Ga-rich region of the binary Ni-Ga system. They refuted the phase “NiGa_4_” crystallizing in a gamma-brass structure and substantiated the phase “Ni_3_Ga_7_” crystallizing in an Ir_3_Ge_7_ structure instead. In addition, they discovered unidentifiable XRD reflections, coinciding with those reported by Yamazaki et al. [15], in alloys with nominal compositions exceeding 80 at% Ga, and attributed the additional reflections to a new phase. Attempts to prepare a single-phase alloy or a single crystal of this new phase for crystal structure solving were unsuccessful. Therefore, this new phase, NiGa_5_, was assumed to be isostructural to PdGa_5_ with Ni replacing Pd, and the lattice constants and the tunable atomic position were determined by refining the powder XRD pattern of the specimens containing the additional reflections. Unfortunately, the phase was not found in the microstructure for microarea chemical analysis, and therefore the composition of the IMC was tentatively assumed to be NiGa_5_. The authors also stated that whether NiGa_5_ is a stable equilibrium phase is inconclusive because the as-quenched specimen had greater intensity of the phase than the annealed specimen.

Doyoung Lee et al. [16] prepared a Ni-Ga diffusion couple at temperatures from 250 to 350 °C followed by 2 min of air cooling and subsequent −20 °C freezing. The authors labelled two layers of IMCs in the cross-section: NiGa_x_ on the Ga-side and Ni_3_Ga_7_ on the Ni side. While the thickness of Ni_3_Ga_7_ increased with reaction time, that of NiGa_x_ did not show a clear tendency to change. Scattered values of composition of this NiGa_x_ phase, 85~91 at%, were reported in the study using EDX analysis. The authors found the XRD patterns of Ga-etched specimens inclusive of Ni_3_Ga_7_ and Ni reflections but exclusive of NiGa_x_ reflections. The authors attributed the absence of the reflection of NiGa_x_ to the nanocrystallinity or amorphism of the formed NiGa_x_.

## 3. Materials and Methods

**Figure 1:**
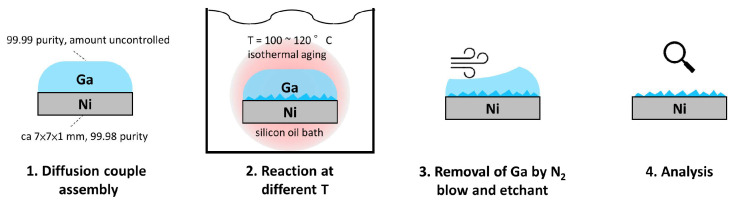
The flow chart of the sample preparation.

The bulk diffusion couples were prepared by assembling pure end members, Ni (99.98%, Goodfellow, Huntingdon, UK) and Ga (99.99%, Super Spark International Co., Taipei, New Taipei City, Taiwan). The nickel foil was cut into 0.8 cm square pieces and polished down to 1 μm diamond. The gallium was heated to melt in a water bath at 40 °C and placed on top of the nickel foil pieces. The assembled diffusion couples were then transferred into a circulating silicone oil bath for isothermal heat treatment with a controlled temperature stability of ±0.4 °C. The heat treatment was performed at 100.1, 110.1, 112.4, 115.1, and 117.4 °C for 168 h (7 days). Additional couples were annealed at 110.1 for 24 h (1 day) and 28 days for the metastability test. After heat treatment, the couple was taken from the bath, and, immediately, most of the molten gallium was blown away by using a nitrogen spray gun; the residual gallium was etched away in a solution of methanol–HCl (4% vol.) mixture, to prevent any possible IMC formation during storage at room temperature and to reveal the interdiffusion zone for reflection XRD analysis. The schematic plot of the sample preparation procedure is presented in Figure 1.

For phase identification, a 2θ-ω scan of X-ray diffraction (XRD) in reflection geometry was carried out on a diffractometer (TTRAX III, Rigaku, Tokyo, Japan) equipped with a CBO unit and a graphite monochromator. The specimens were placed on the thin film attachment with the interdiffusion zone facing the radiation. The radiation source was Cu K_α_ generated at 50 kV voltage and 300 mA current electron bombardment and collimated into a parallel light in the CBO unit.

Specimens to be examined by a scanning electron microscope and electron probe microanalyzer were embedded in epoxy resin (Epofix, Struers, Ballerup, Denmark) and the cross-sections were prepared following the conventional metallographic method. The final step was polishing with Buehler MasterPrep suspension against the porous neoprene cloth.

For quantitative analysis of the elemental composition, FE-EPMA (JEOL, Tokyo, Japan, JXA-8530F Plus) was employed to determine the composition of the obtained IMCs using built-in ZAF correction and applying GaN and Ni as the standards. The electron probe was set at 15 kV voltage and 20 nA current. The intensity of peak, upper background, and lower background were measured for 10, 5, and 5 s, respectively. Metallographic observation was performed under an optical microscope (Axio Scope A1, Zeiss, Jena, Germany) and an SEM (SU5000, Hitachi, Tokyo, Japan) with EDX installed (XFlash 6-60, Bruker, Billerica, MA, USA). The average thickness of each IMC layer was calculated by dividing the cross-sectional IMC area by the length of field of view.

## 4. Results and Discussion

**Figure 2:**
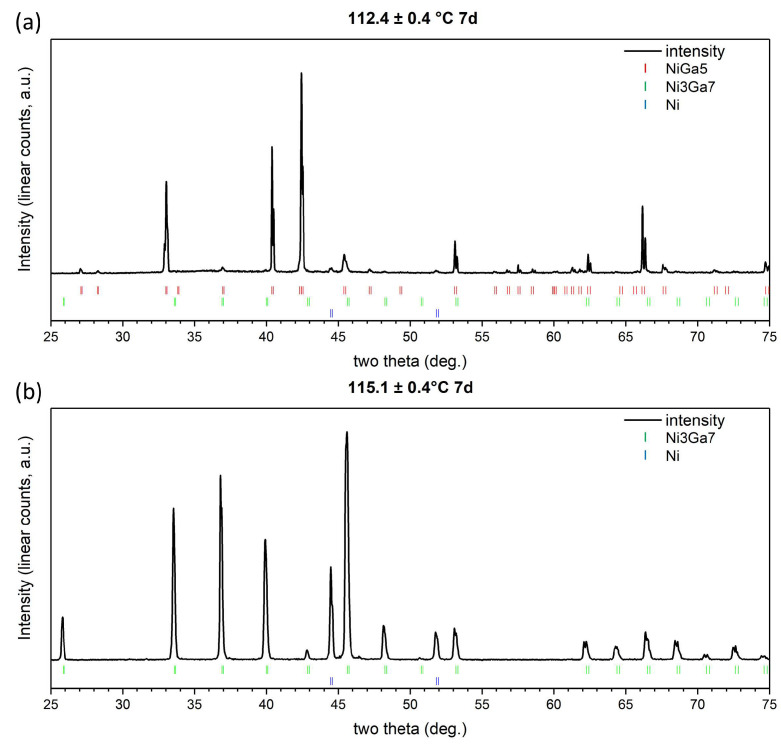
XRD pattern of diffusion couples annealed at (**a**) 112.4 and (**b**) 115.1 °C.

To identify the species of the IMCs formed in the interdiffusion zone, the experimentally obtained XRD patterns were compared with the theoretical diffraction patterns of possibly formed phases and the substrate. Crystal structure data (Table 1) of Ni_3_Ga_7_ [17], NiGa_5_ [14], and Ni [18] were input into VESTA software (Ver. 3.5.7) [19] to simulate the theoretical PXRD peak positions. The incident wavelengths were set as 0.154059 nm (Cu K_α1_) and 0.154432 nm (Cu K_α2_). Then, the simulated reflection positions were stacked at the bottom of the experimental patterns, as shown in Figure 2. For specimens isothermally annealed at 110.1 and 112.4 °C (Figure 2a), reflections of the NiGa_5_ phase can be identified. In contrast, the NiGa_5_ phase is absent in specimens isothermally annealed at 115.1 (Figure 2b) and 117.4 °C. The result suggests that the peritectic reaction $L+Ni_{3}Ga_{7}\to NiGa_{5}$ exists in the binary system and, considering the temperature stability of the heating facility, the temperature of the invariant reaction is in between 112.0 and 115.5 °C.

Clemens Schmetterer et al. [14] conducted a non-ambient XRD experiment on a mixture containing the NiGa_5_ phase along with other binary Ni-Ga phases. They reported that the intensity of the phase decreased from 100 to 108 °C and disappeared at 125 °C; hence, the peritectic temperature was anticipated to lie between 100 and 108 °C. The present study used the diffusion couple techniques and obtained a more precise temperature range of the peritectic reaction.

**Figure 3:**
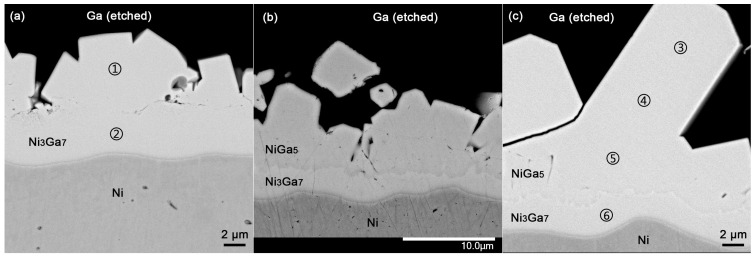
Micrographs of couples with positions of EPMA point analysis indicated: (**a**) annealed at 115.1 °C for 7 days; (**b**) annealed at 112.4 °C for 7 days; (**c**) annealed at 100.1 °C for 7 days. The numbers in the figures indicate the position of EPMA point analysis, and the corresponding results are presented in Table 2.

In order to double-confirm the grown phases metallographically and perform microarea composition analysis on each phase, the cross-sections of the diffusion couples were prepared and shown in Figure 3a–c. The NiGa_5_ phase was absent in the diffusion couple annealed at 115.1 °C and was present in the diffusion couple annealed at 112.4 °C, which is consistent with the XRD results. The interphase boundaries are shown by a light line adjacent to a dark line in the SEM images due to the edge effect. EPMA point analysis was conducted at the indicated positions and is summarized in Table 2. The composition gradient of the NiGa_5_ phase along the interdiffusion direction was small, suggesting a narrow homogeneity range of this phase above 100 °C. It is noticed that the measured compositions of NiGa_5_ and Ni_3_Ga_7_ are slightly more Ni-rich than the stoichiometry. This is because the K-line X-rays of Ga-excited Ni atoms of neighboring phases contain more Ni, causing additional boundary fluorescence of Ni K lines. This manifests in the fact that the harvested intensity and the un-normalized total weight percent increase as the site of point analysis approaches the Ni substrate. Therefore, it is believed that the IMCs are of stoichiometric compositions and that the measured off-stoichiometric compositions are due to the parasitic boundary fluorescence.

**Figure 4:**
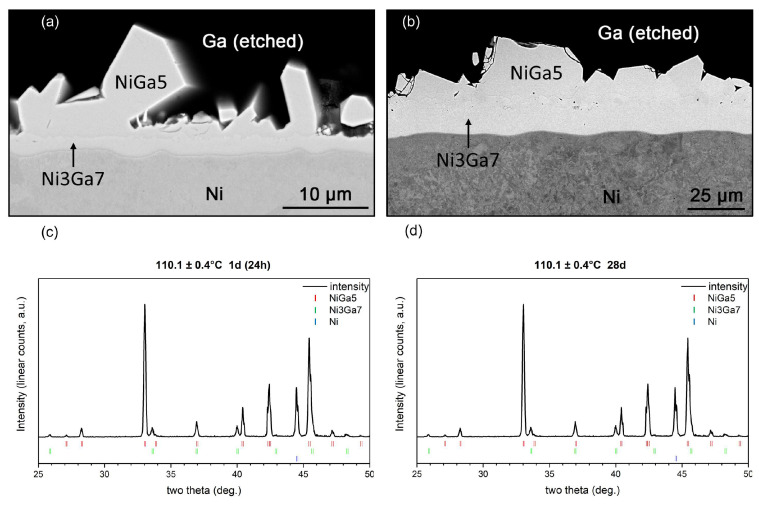
Micrographs of couples annealed at 110.1 °C for (**a**) 24 h and (**b**) 28 d, and (**c**,**d**) the corresponding XRD patterns.

**Figure 5:**
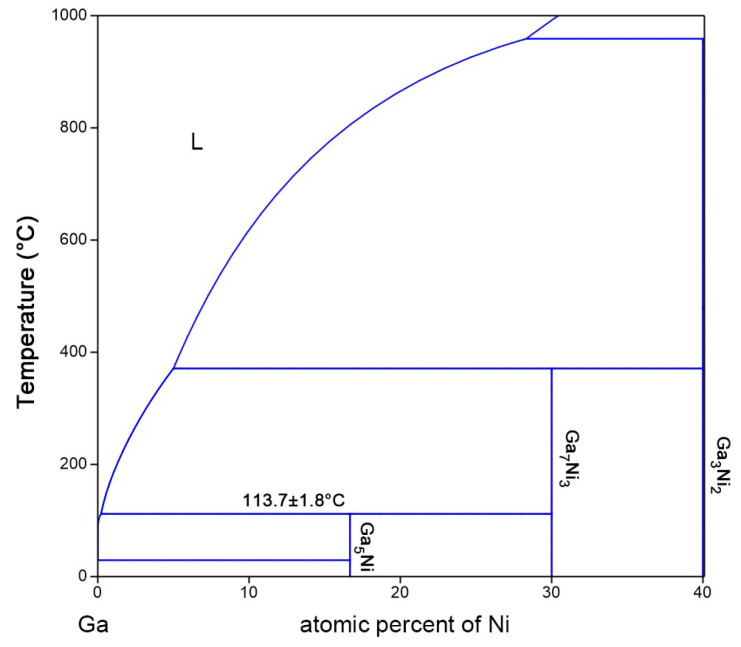
The proposed phase diagram with the addition of the Ga_5_Ni phase. The uncertainty of the peritectic temperature is from the temperature stability of the silicon oil bath and the temperature difference of heat treatment performed on the diffusion couples. L denotes liquid.

To determine the metastability of the NiGa_5_ phase, additional diffusion couples were prepared at 110.1 °C for 1 day and 28 days. Both diffusion couples showed two IMCs in the microstructure; one was NiGa_5_ (83.4 ± 0.8 at% Ga) and the other was Ni_3_Ga_7_ (68.4 ± 1.1 at% Ga), as indicated by multiple EDX point analysis. The XRD patterns also confirmed the species of the IMCs (Figure 4). Compared to the faceted NiGa_5_ layer of an average thickness of c.a. 2.6 um on the 1-day-annealed specimen, the NiGa_5_ layer on the 28-day-annealed specimen grew to an average thickness of 14.8 um as opposed to thickness decrease or decomposition. This fact signifies that NiGa_5_ is indeed a stable phase that does not decompose after prolonged heat treatment. Combined with the result of the product of the diffusion couples at different temperatures, it is suggested that the peritectic reaction $L+Ni_{3}Ga_{7}\to NiGa_{5}$ be drawn in the binary equilibrium phase diagram, as demonstrated in Figure 5.

## 5. Conclusions

The Ga-rich low-T part of the Ni-Ga phase equilibrium has been investigated due to the lack of experimental literature regarding the NiGa_5_ phase. The homogeneity range of the NiGa_5_ phase, its peritectic temperature, and the evidence of its stability have been given. In addition, the present work demonstrated the usefulness of the diffusion couple technique in constructing low-temperature part of phase diagrams, particularly when there is difficulty equilibrating an ingot quenched from the liquid state due to a low diffusion rate or a thick primary phase.

## Figures and Tables

**Table 1:** Selected crystallographic information of the chosen structure for each phase.

Phase	Space Group	Lattice Constants (Å)	Reference
NiGa_5_	I4/mcm	a = 6.3128, c = 9.7217	[14]
Ni_3_Ga_7_	Im3m	a = 8.4285	[17]
Ni	Fm-3m	a = 3.525	[18]

**Table 2:** EPMA point analysis result of positions indicated in Figure 3.

Diffusion Couple	Site	Ga (at%)	Ni (at%)	Σ mass %	Phase
115.1 ± 0.4 °C 7 d	①	69.5	30.5	99.0	Ni_3_Ga_7_
	②	68.7	31.3	101.0	Ni_3_Ga_7_
100.1 ± 0.4 °C 7 d	③	81.2	18.8	99.4	NiGa_5_
	④	81.1	18.9	99.6	NiGa_5_
	⑤	81.0	19.0	101.6	NiGa_5_
	⑥	69.6	30.4	101.4	Ni_3_Ga_7_

## Data Availability

Data available upon reasonable request.
